# Bronchial artery pseudoaneurysm presenting with hemoptysis and hemothorax after pulmonary lobectomy: a case report and literature review

**DOI:** 10.1186/s13019-024-03328-z

**Published:** 2024-12-31

**Authors:** Wei-Hao Zhang, Cheng-Yan Jin, Xiao Qi, Guang-Xin Zhang

**Affiliations:** 1 The Second Clinical Medical School, Jilin University, Changchun, Jilin 130041 PR China; 2 Department of Thoracic Surgery, The Second Hospital of Jilin University, Changchun, Jilin 130041 PR China; 3 No. 4026, Yatai Street, Nanguan District, 13840314134 Changchun City, Jilin Province China

**Keywords:** Angiography, Vascular trauma, Peripheral artery disease (PAD), Interventional radiology, Vascular imaging/diagnostics

## Abstract

**Background:**

Bronchial artery pseudoaneurysm is a rare vascular disorder, and cases of bronchial pseudoaneurysms reported after lung surgery are even rarer. The number of reported cases is very limited due to the unclear pathogenesis, lack of diagnostic criteria and treatment guidelines, and nonspecific clinical manifestations.

**Case presentation:**

The paper reports a case of a patient with primary lung adenocarcinoma who developed hemoptysis, chest and back pain, and right hemothorax after lobectomy. Due to the lack of experience in treatment, we suspected that the above symptoms were caused by postoperative broncho-vascular fistula. The second thoracotomy and bronchoscopy examination did not reveal any obvious hemorrhage or fistula, and a subsequent bronchial arteriography confirmed the presence of a pseudoaneurysm in the right main bronchial artery. Later, the patient underwent transcatheter bronchial artery embolization and was followed up for half of a year after the operation without experiencing any of the previously mentioned symptoms.

**Conclusions:**

The case reports the successful cure of bronchial artery pseudoaneurysm after pulmonary lobectomy through bronchial artery embolization.

## Background

Bronchial artery aneurysm (BAA) is a rare occurrence, accounting for only 1% of all elective bronchial arteriography cases, and bronchial artery pseudoaneurysm (BAP) is even rarer [1]. The etiologies of bronchial aneurysms and pseudoaneurysms include trauma, bronchopulmonary infections, pulmonary malignancies, coagulation disorders, and connective tissue diseases. BAP may be asymptomatic as well as can cause hemoptysis, chest and back pain, mediastinal blood accumulation, dyspnea, and even hemorrhagic shock after rupture [2, 3]. Due to its location, size, shape, and whether it is entrapped in the wall, differentiating BAP from other conditions such as broncho-vascular fistula (BVF) and mediastinal tumors by non-invasive imaging examinations can be challenging. Subsequently lots of cases are only detected incidentally [4] and the Angiography is the most sensitive method for qualitatively diagnosing BAP and determining hemodynamic changes [5].

## Case presentation

### Case description

The hospital admitted a male patient in his early 50s. with a pulmonary nodule discovered during a physical examination one month prior. The patient had a 5-year history of hypertension and denied any family history of hereditary or related disorders, as well as any history of surgery or trauma. The patient’s chest CT indicated a malignant tumor, leading to a wedge resection of the upper lobe of the right lung by VATS (video-assisted thoracic surgery). Intraoperative frozen section pathology confirmed invasive adenocarcinoma, resulting in further resection of the upper lobe of the right lung. Intraoperative exploration of the horizontal fissure was rudimentary. To achieve the aim of radical surgery, the horizontal fissure was opened using a cutting suture. Surgical staplers were used to isolate the upper pulmonary vein, the cusp branch of the pulmonary artery, the anterior and posterior branches of the pulmonary artery, and the bronchus of the upper lobe, as well as the lymph node dissection was performed (mediastinal ND 4, 7, 8 ,9 and intrapulmonary ND 10, 11, 12). The patient had a good recovery and was discharged from the hospital 5 days after the operation. After being discharged, the patient did not exhibit any signs of fever, cough, sputum, or discomfort after physical activity.

**Fig. 1:**
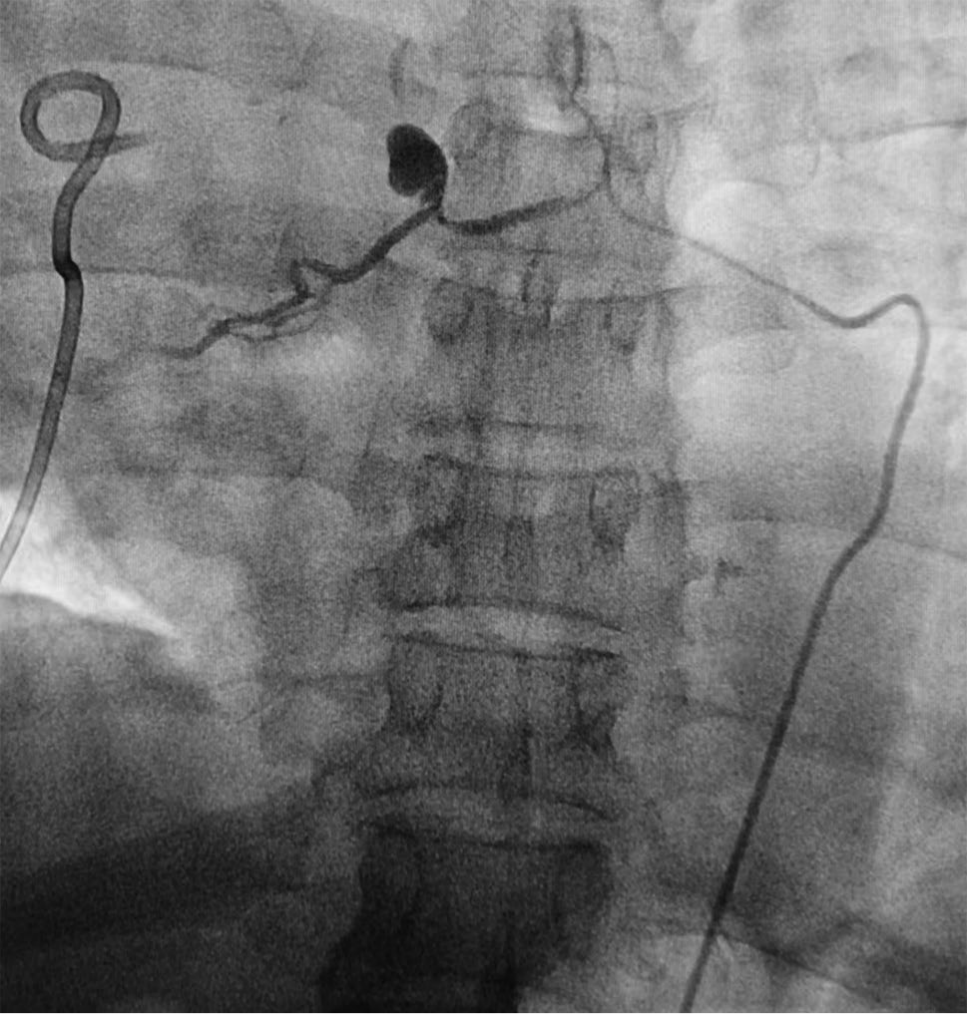
A large bronchial artery is seen emanating from the thoracic aorta to the right. The bronchial artery pseudoaneurysm stain in the middle of the main artery is observed, which is of the mediastinum type

**Fig. 2:**
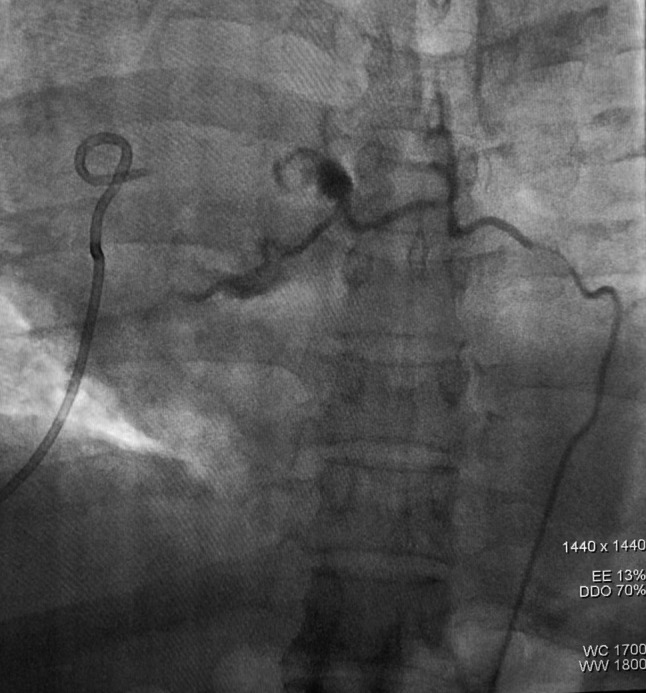
The distal end of the bronchial artery pseudoaneurysm was gradually embolized to the main bronchial artery on the right side. Upon reexamination of the angiography, occlusion of the right bronchial artery was observed

**Fig. 3:**
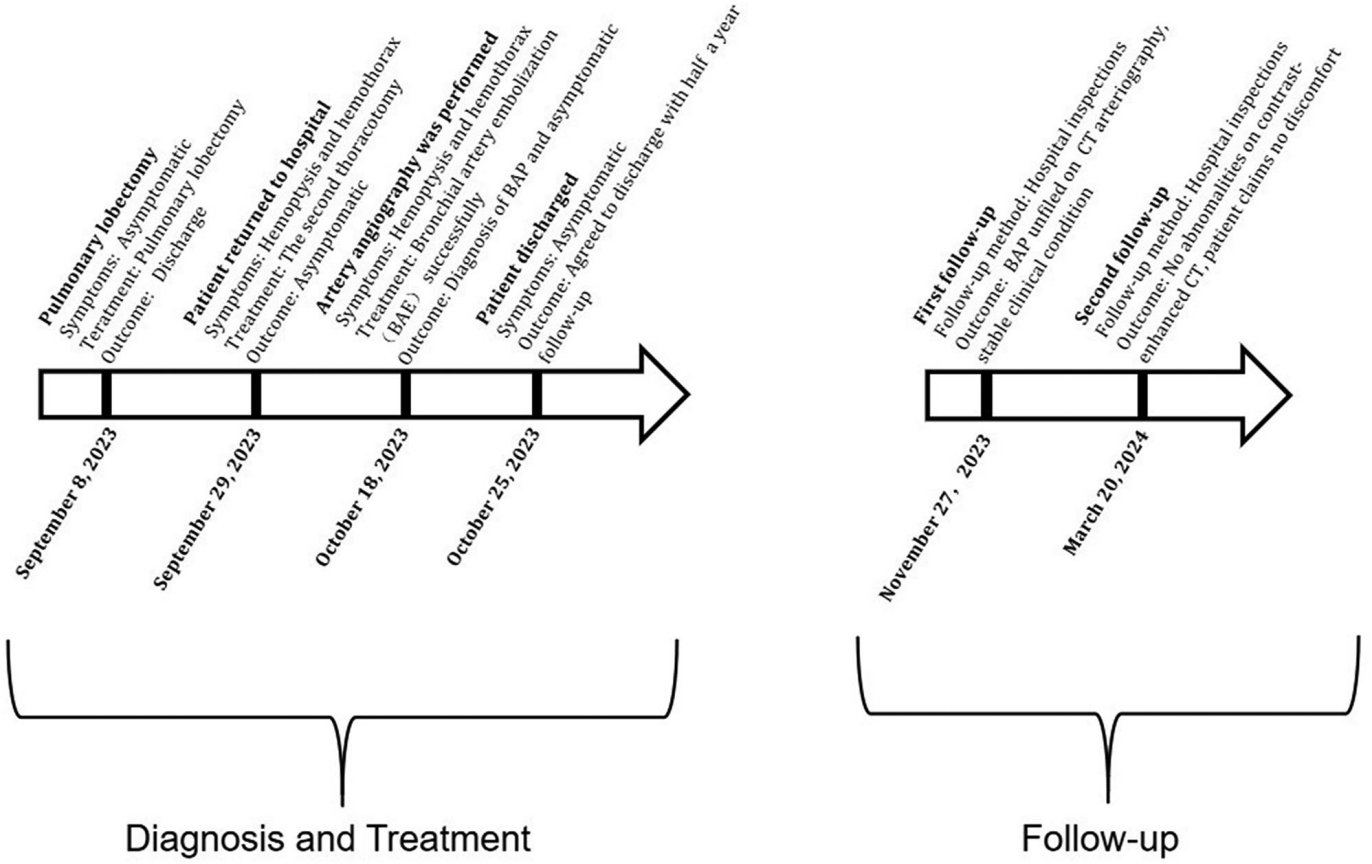
The timeline for the diagnosis, treatment, and follow-up of this case

### Diagnostic assessment

On the 15th day post-discharge, the patient experienced hemoptysis (200 ml) and had an elevated Leukocyte count of 15.6 × 10^9/L, Neutrophil count of 12.11 × 10^9/L as well as a decreased Erythrocyte count of 3.89 × 10^12/L, Hemoglobin level of 118 g/L. The chest CT indicates a significant accumulation of fluid in the pleural cavity on the right side and an enclosed accumulation of fluid around the tracheal stump in the upper lobe of the right lung. A closed pleural drainage procedure was performed, resulting in the removal of a large amount of bloody fluid (1000 ml), and the drainage tube was immediately clamped shut. The drainage tube was immediately clamped off after 1000 ml of fluid was drained. We suspected it was progressive hemothorax caused by postoperative bronchial stump arterial hemorrhage that allowed the patient to develop hemorrhagic shock. Then the urgent thoracotomy by VATS was performed. No hemoptysis caused by bronchial stump fistula was observed. The upper lobe bronchial stumps, vascular segments and cut edges of the lung tissues were carefully examined, but no hemorrhage was detected. After the surgery, the patient recovered without any discomfort and did not experience hemoptysis or chest pain. During the second thoracic exploration, no bleeding points were observed. This may be due to the negative to positive intra-thoracic pressure after opening the chest. The patient’s condition was stable after the operation. However, the patient’s family refused further examination, so no further treatment was administered. We believe that the postoperative BVF caused the aforementioned symptoms.

### Treatment

**Fig. 4:**
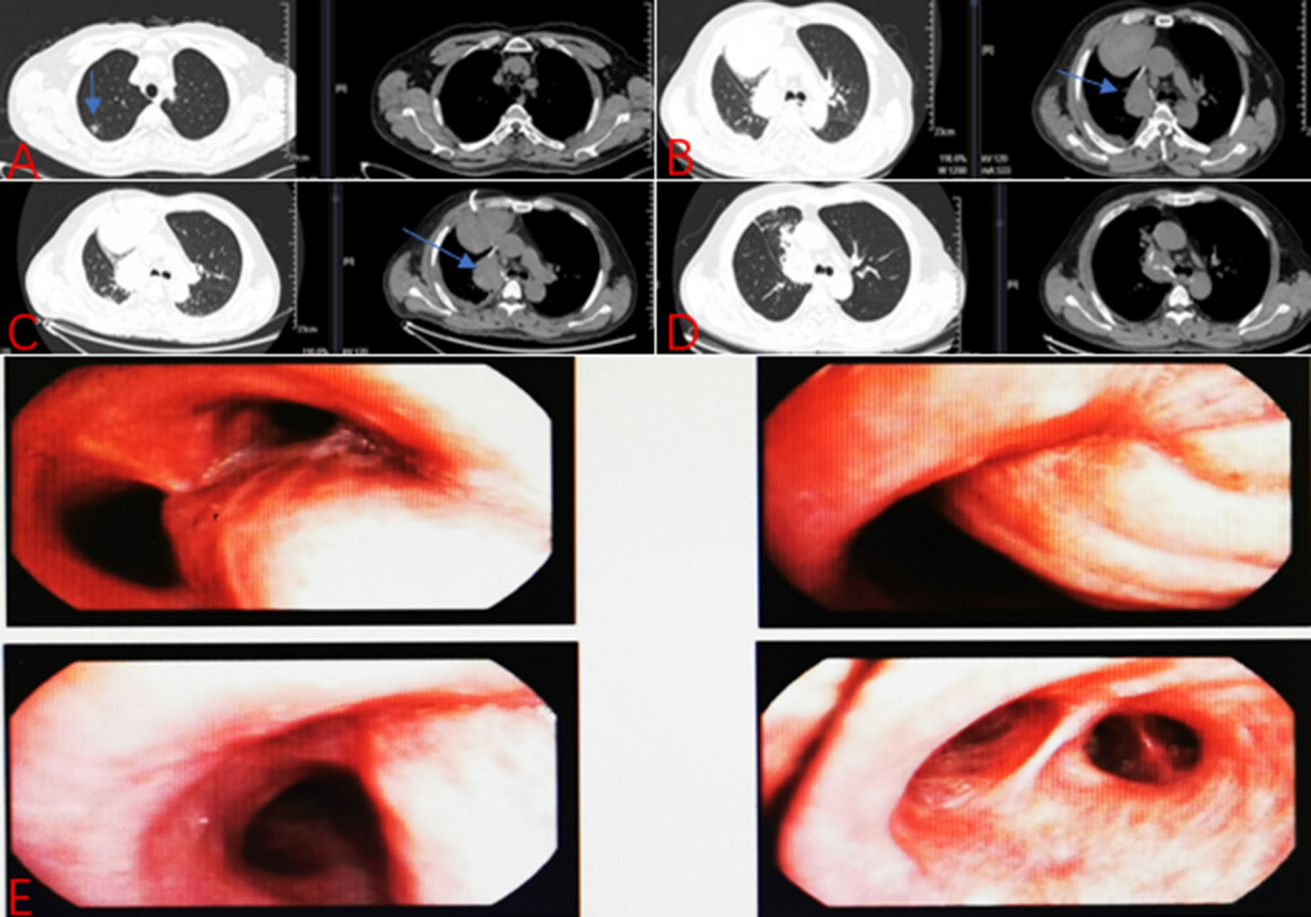
Radiographic findings of the patient. **A**: CT scan conducted prior to lobectomy revealed the presence of a mixed ground-glass nodule within the upper lobe of the right lung (as shown by the blue arrow). **B**: CT scan after the patient presented with hemoptysis for the first time following lobectomy, which revealed encapsulated hemoptysis at the bronchial stump (as shown by the blue arrow). **C**: CT scan of the patient exhibited the aforementioned symptoms for a second time, presenting with an encapsulated hematoma in a location that was almost identical to the previous occurrence (as shown by the blue arrow). **D**: CT scan conducted one month after the angiography demonstrated a notable reduction in blood and fluid absorption. **E**: The bronchoscopy examination revealed no discernible abnormality

On the thirteenth day after the second thoracotomy, the patient experienced hemoptysis again, with approximately 100 ml of blood, accompanied by severe chest and back pain. The repeated chest CT scan revealed a peripheral encapsulated pleural effusion around the bronchial stump of upper right lobe, which was drained through puncture of the hemorrhagic fluid. Bronchoscopy examination indicated that there were no active hemorrhage points in the bronchial stumps of the right upper lobe of the right lung or in the remaining lung tissues (Fig. 4). Then we asked the Interventional radiologist to performed the angiography and occluded the bronchial artery pseudoaneurysm. The result showed a tortuous and dilated bronchial artery with staining indicative of a pseudoaneurysm in its trunk (Fig. 1). The occlusion was confirmed through imaging review (Fig. 2). Blood flow in the target artery was completely stopped. The patient was followed up for six months after the operation without experiencing any further discomfort. Figure below illustrates the timeline for the diagnosis, treatment, and follow-up of this case (Fig. 3).

## Discussion

A pseudoaneurysm is a collection of the thrombus that has been organized through fibrosis of the thrombus or surrounding structures. It can be caused by trauma, a ruptured aneurysm, or a postoperative anastomotic fistula. The bronchial artery of the patient was observed to be dilated and tortuous with weakened arterial wall strength by digital subtraction angiography (DSA). During the first operation, the artery maybe suffered non-penetrating rupture due to trauma. The damaged artery and perivascular tissues encapsulated the rupture, forming a perfused sac-like structure that communicated with the vessel’s lumen. Hemorrhage occurred after the rupture of the pseudoaneurysm [6]. Bronchial tubes and arteries share a common connective tissue sheath. Blood from ruptured aneurysms can enter the bronchial tubes directly, leading to hemoptysis [7], which presents as persistent or intermittent hemoptysis. Aneurysms typically present with severe, lacerating chest pain radiating to the back and epigastric region, and can also affect the pleural cavity and mediastinum. Prompt intervention is required after rupture of a pseudoaneurysm to prevent the formation of a hemothorax and subsequent hemorrhagic shock [4].

Hemoptysis with thoracic bleeding can also occur in bronchial stump vascular micro-fistula after lobectomy [8]. If BVF is suspected, bronchoscopy examination should be performed [9]. The patient presented with symptoms of hemorrhagic shock and was bleeding heavily. However, we did not observe any obvious fistulae or pseudoaneurysm formation.

The diagnosis of BAP is typically made using DSA, which has been shown to be highly sensitive and accurate. In fact, a study by Habib N reported a 100% sensitivity rate for DSA in detecting BAP cases [5]. Additionally, DSA allows for real-time assessment of contrast medium filling in the lesion [10], making it the gold standard for BAP diagnosis. Bronchial artery embolization (BAE) is a frequently used treatment for pseudoaneurysms, which has a high cure rate. In recent years, it has become the first-line treatment for them [11].

A review of previously literatures (Table 1) revealed that the most common clinical manifestations of this condition included hemoptysis (especially intrapulmonary hemoptysis) and rupture. It is most prevalent in individuals aged 40 to 70 years. The primary etiologies were identified as congenital, post-traumatic, and post-inflammatory. The majority of cases were believed to have a pulmonary and bronchial infection as their etiology, with some cases thought to be due to bronchial aneurysm degeneration. BAE is the primary method utilized for the treatment of pseudoaneurysms originating from bronchial arteries. Nevertheless, open surgical repair, such as sternotomy or lateral sternotomy, represents an alternative option when endovascular treatment is not a viable option.

**Table 1:** Cases of bronchial pseudoaneurysms in the literature

Reference	Patient	Symptoms	Cause	Treatment	Outcome
Patel [1]	A 10s female	Hemoptysis	Pneumonia	Embolization	Cure
Van Hove P [3]	An 80s female	Acute retrosternal pain	Pneumonia	Embolization	Cure
Kaufman [4]	A 60s male	Repture	Bronchial aneurysm degenerration	Embolization	Cure
Nguyen [12]	A 60s female	Repture	Bronchial thermoplasty	Embolization	Cure
Izaaryene [13]	A 30s male	Dysphagia, dry cough	Incidental	Embolization	Cure
Urlings [14]	A 70s male	Hemoptysis	Lung Cancer	Embolization	Cure
Raboso [15]	A 60s female	Hemoptysis	Postlobectomy	Embolization	Uncured
Koirala [16]	A 40s male	Repture	Incidental	Embolization	Cure
Ghonge [17]	A 40s male	Asympomatic	Tuberculosis	Embolization	Cure
Braithwaite [18]	A 50s male	Hemoptysis	Pneumonia	Embolization	Cure
Recalde-Zamacona [19]	A 60s male	Hemoptysis	EBUS-FNA^a^	Embolization	Cure
Kabilan [20]	A young male	Hemoptysis	Tuberculosis	Embolization	Cure
Eric Yu Wei Lo [21]	A 50s male	Asympomatic	Using warfarin therapy	Embolization	Cure
Budacan AM [22]	A 40s male	Cough and haemoptysis	Traumatic	Embolization	Cure

a: EBUS-FNA = endobronchial ultrasound fine needle aspiration

Reviewing the case, we performed right upper lobe resection and lymph node dissection. During the operation, we firstly cleaned ND 9–11, and then cut each blood vessel and the bronchus with surgical staples to remove the upper lobe of the right lung. Preoperative chest CT examination of the patient found no obvious abnormalities in the pulmonary blood vessels and bronchus, nor was there any recent history of infection or oral anticoagulant drugs. Furthermore, due to the blood clots were identified at the bronchial stump during the second thoracotomy by VATS, and subsequent chest CT examinations indicated the formation of wrapped hemoptysis at the bronchial stump in response to each episode of hemoptysis of the patient (Fig. 4), we hypothesize that the surgery is the injury resulting in BAP. However, due to our lack of experience in the diagnosis and treatment of BAP as well as the small size of the pseudoaneurysm and the limitation of chest CT examination itself, we have not detected the existence of pseudoaneurysm until angiography. Furthermore, embolization of the bronchial artery may potentially elevate the risk of bronchial stump fistula, which was not identified prior to the arterial embolization procedure. The patient is still being monitored regularly following the surgical procedure. The treatment process was arduous, yet this is the inaugural case of a BAP being successfully cured following lobectomy.

## Conclusion

During the patient’s second open chest exploration, no active bleeding points were observed. Similarly, bronchoscopy did not reveal any active bleeding points. The problem was identified in a timely manner through DSA after a second episode of bleeding hemoptysis and thoracic effusion. In this case, if bronchial arteriography performs earlier, it could prevent the patient from experiencing hemoptysis again. This can reduce psychological trauma, shorten hospitalization time, and lower treatment costs. The study serves as a reminder to be alert for the possibility of a stump pseudoaneurysm in patients who present with hemoptysis and pleural effusion in the postoperative period.

## Data Availability

No datasets were generated or analysed during the current study.

## Abbreviations

BAP: bronchial artery pseudoaneurysm
BVF: broncho-vascular fistula
BAE: bronchial artery embolization
VATS: video-assisted thoracic surgery
DSA: digital subtraction angiography
ND: lymph node
